# mHealth App to Promote Healthy Lifestyles for Diverse Families Living in Rural Areas: Usability Study

**DOI:** 10.2196/60495

**Published:** 2025-02-11

**Authors:** Alejandra Perez Ramirez, Adrian Ortega, Natalie Stephenson, Angel Muñoz Osorio, Anne Kazak, Thao-Ly Phan

**Affiliations:** 1 Center For Healthcare Delivery Science Nemours Children's Health Wilmington, DE United States; 2 Center for Behavior Intervention Technologies Northwestern Feinberg School of Medicine Northwestern University Evanston, IL United States; 3 Center for Health Delivery Innovation Nemours Children's Health Wilmington, DE United States; 4 Department of Pediatrics Sidney Kimmel Medical College Thomas Jefferson University Philadelphia, PA United States

**Keywords:** obesity, user testing, mHealth, mobile health, Spanish, child, rural population

## Abstract

**Background:**

Mobile Integrated Care for Childhood Obesity is a multicomponent intervention for caregivers of young children with obesity from rural communities that was developed in collaboration with community, parent, and health care partners. It includes community programming to promote healthy lifestyles and address social needs and health care visits with an interdisciplinary team. A digital mobile health platform—the Healthy Lifestyle (Nemours Children’s Health) dashboard—was designed as a self-management tool for caregivers to use as part of Mobile Integrated Care for Childhood Obesity.

**Objective:**

This study aimed to improve the usability of the English and Spanish language versions of the Healthy Lifestyle dashboard.

**Methods:**

During a 3-phased approach, usability testing was conducted with a diverse group of parents. In total, 7 mothers of children with obesity from rural communities (average age 39, SD 4.9 years; 4 Spanish-speaking and 3 English-speaking) provided feedback on a prototype of the dashboard. Participants verbalized their thoughts while using the prototype to complete 4 tasks. Preferences on the dashboard icon and resource page layout were also collected. Testing was done until feedback reached saturation and no additional substantive changes were suggested. Qualitative and quantitative data regarding usability, acceptability, and understandability were analyzed.

**Results:**

The dashboard was noted to be acceptable by 100% (N=7) of the participants. Overall, participants found the dashboard easy to navigate and found the resources, notifications, and ability to communicate with the health care team to be especially helpful. However, all (N=4) of the Spanish-speaking participants identified challenges related to numeracy (eg, difficulty interpreting the growth chart) and literacy (eg, features not fully available in Spanish), which informed iterative refinements to make the dashboard clearer and more literacy-sensitive. All 7 participants (100%) selected the same dashboard icon and 71% (5/7) preferred the final resource page layout.

**Conclusions:**

Conducting usability testing with key demographic populations, especially Spanish-speaking populations, was important to developing a mobile health intervention that is user-friendly, culturally relevant, and literacy-sensitive.

## Introduction

Childhood obesity persists as a significant public health concern in the United States [1,2]. Notably, 1 in every 5 children in the United States lives with obesity. Obesity in childhood is linked to increased risks of comorbid and future health conditions such as diabetes, cardiovascular diseases, cancer, and psychosocial concerns, leading to increased health care costs and utilization [1,3]. This is especially the case for children from communities of color, communities with fewer resources, and rural communities, who continue to have the highest rates of obesity [2]. Challenges in affording and accessing nutritious food, accessing safe spaces for physical activity, having transportation to get to health care services, and managing family stressors are a few of the many systemic barriers that perpetuate disparities in childhood obesity [4,5]. While lifestyle behavior interventions are first-line treatments for improving health behaviors and obesity outcomes, most available interventions do not address these systemic barriers and are often not effective when implemented in the communities with the greatest need [6,7]. Thus, more studies are needed that consider the needs and preferences of diverse patient families when designing an intervention.

Digital interventions for the treatment of pediatric obesity can increase the convenience and access to treatment through the removal of common barriers such as financial, travel, and time constraints [8,9]. A recent systematic review and meta-analysis revealed that digital interventions for the treatment of pediatric obesity have a significant yet small effect on BMI *z* scores [10]. Still, there are important limitations to digital interventions that require consideration, especially regarding their uptake and implementation. For one, digital interventions are subject to high nonusage attrition such as low uptake and disengagement [11,12]. Although some digital interventions for the treatment of pediatric obesity have included diverse samples in their studies, there remain concerns about their cultural sensitivity [10]. For example, most of these interventions do not include content related to systemic barriers, social drivers of health, or community resources [13]. Rurality or place of residence is also not often reported or considered when implementing digital interventions [14]. In addition, there is only 1 published study of a digital intervention for pediatric obesity available in Spanish, which is a major gap given the prevalence of Hispanic or Latino children with overweight or obesity in the United States [5,15,16]. Therefore, there is a need to incorporate human-centered design principles into the design of digital interventions, such as partnering with target users to enhance the usability of these interventions. This way, digital intervention designers can maximize engagement by building an intervention that centers on the needs and preferences of its users. Key principles of human-centered design include but are not limited to the active involvement of users with lived experiences relevant to the intervention in design activities as well as iteratively refining and updating intervention features based on user feedback [17].

**Figure 1:**
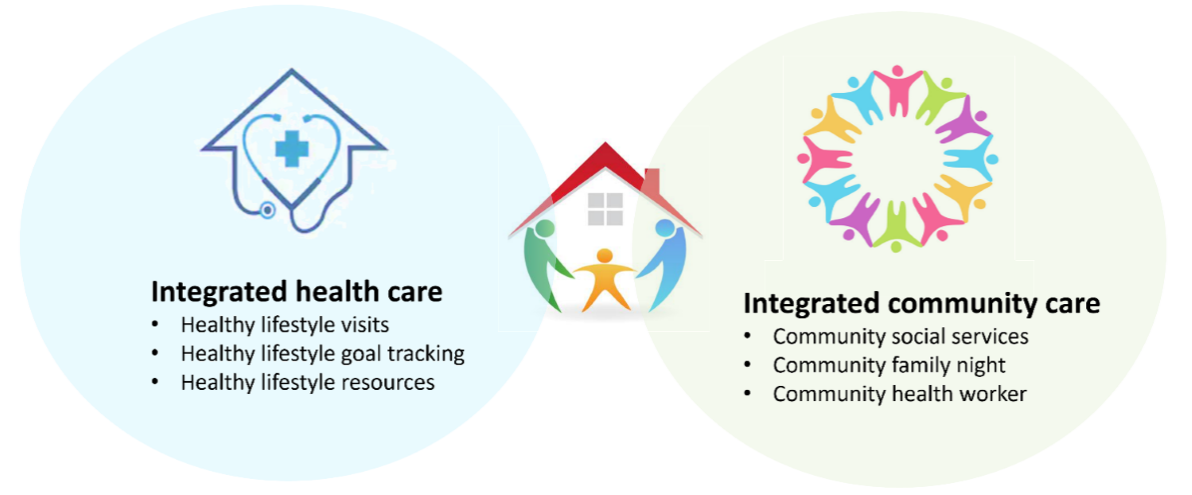
Mobile Integrated Care for Childhood Obesity (MICCO) care model, developed by a research team for families of young children with obesity.

Drawing on the advantages of using a digital intervention, a digital mobile health (mHealth) platform was included as part of a larger, multicomponent intervention called Mobile Integrated Care for Childhood Obesity (MICCO). MICCO was designed for caregivers of children with obesity from rural communities (Figure 1). MICCO combines evidence-based interdisciplinary obesity treatment, monthly community-based programming for families to engage in physical activity and have a healthy affordable meal together, and community health worker outreach to address psychosocial needs and barriers to achieving lifestyle goals. Importantly, MICCO was developed with the input of a Council of Parent Partners, which included 6 parents of children with obesity from rural communities, and a Council of Community Partners, which included 5 primary care providers from clinics serving rural communities and 4 community organizations serving rural communities. These Councils met routinely by videoconference throughout the entirety of the study to provide valuable feedback on the development and implementation of the MICCO intervention, drawing on their knowledge about the needs of families of children with obesity and resources in the community to support families.

As an additional component of MICCO, a custom Healthy Lifestyle dashboard on a mobile app linked to the Nemours Children’s Health electronic health record (EHR; Nemours App [Nemours Children’s Health]) was built, with input from interdisciplinary specialists from the Nemours Healthy Weight and Wellness Clinic and Council of Community Partners and in collaboration with app designers from the Nemours Center for Health Delivery Innovation. Psychoeducational tools based on social cognitive theory [18], behavior change techniques [19], and the socioecological model of obesity [20] were included on the dashboard, including a self-monitoring function to track daily progress toward lifestyle goals formed during treatment visits and culturally relevant videos to promote positive parenting and healthy lifestyle behaviors. The video resources, including 6 that were created specifically for the MICCO intervention, provided content related to mood and behavior, physical activity, and healthy eating; were available in English and Spanish; and featured families and health care providers of diverse backgrounds. Through the dashboard, caregivers can also track their child’s weight using an integrated Bluetooth scale, send messages to their health care team, and connect to telehealth appointments. The dashboard also included links to community programs (including healthy lifestyle and social need resources) and monthly reminders about the monthly community-based program. Since many of the patient families with obesity served by Nemours Children’s Health are Hispanic or Latino, the dashboard was developed in English and Spanish. A parallel dashboard was created for health care providers to access data entered by patient families about their goals and weight progress to assist with clinical decision support.

User testing was conducted to test and refine the MICCO Healthy Lifestyle dashboard to enhance the usability of the intervention for families of diverse backgrounds. No hypotheses were made a priori.

## Methods

### Overview

Parents of children with obesity living in rural communities provided feedback on the usability of the Healthy Lifestyle dashboard through Think Aloud testing. Think Aloud testing is a research method applied to an iterative human-centered design process to discover usability problems with a product, incorporate feedback, revise the product, and retest to validate the design. This evidence-based method for obtaining in vivo feedback has been used in the development of digital health interventions [21]. In a typical usability study, the most common usability problems are uncovered after testing with 5 participants [22].

### Sample and Recruitment

Target users of the MICCO intervention are legal guardians of children ages 4-12 years with obesity (BMI percentile ≥95) receiving primary care from 1 of 3 Nemours Children’s Health practices in Delaware serving rural communities. Rurality was determined based on a score of 4 or greater on the US Department of Agriculture 2010 Rural-Urban Commuting Area Codes [23]. Therefore, we recruited participants matching these characteristics for user testing. Legal guardians also had to be able to read English or Spanish and have access to the internet. A primary aim of the study was to incorporate diverse perspectives in refining the Healthy Lifestyle dashboard; therefore, purposeful sampling was used to recruit a diverse group of parents including Spanish-speaking parents of Hispanic or Latino ethnicity and English-speaking parents of Black and White race. A list of potentially eligible participants was pulled from the EHR of Nemours Children’s Health, which is a large pediatric health care system with 12 primary care clinics in Delaware. A total of 196 parents (63 Spanish-speaking and 133 English-speaking) from this EHR list received an SMS text message informing them of the study. In total, 34 parents (15 Spanish-speaking and 19 English-speaking) responded to the text. A research coordinator then followed up with a call to discuss the study and procedures with each parent. Of these, 7 agreed to participate (4 Spanish-speaking and 3 English-speaking), 2 declined after hearing about the study, and the remainder did not respond to any additional contact by the study team. For those who agreed to participate, the research coordinator scheduled a Think Aloud session. Consent was collected at the time of the session before any data collection (refer to Ethical Considerations for details regarding the consent process).

### Data Collection

Think Aloud testing was conducted via video calls through a secure Zoom (Zoom Communications) platform, accessible to participants either on their mobile device or computer. A web link to an interactive, high-fidelity prototype of the Healthy Lifestyle dashboard was shared with participants. The online prototype mirrored the app and allowed participants to easily access the design for Think Aloud testing. The sessions were done through a video call, similar to other studies [24], to increase the convenience for parents of young children and to simulate the environmental context within which the actual mobile app would be experienced. It also allowed for screen recording so the research coordinator could see which parts were easy or hard to navigate.

The testing sessions were video-recorded, and the audio was transcribed. Spanish language recordings were translated and transcribed into English. Rarely, participants encountered minor technical issues with Zoom, such as slow connectivity or difficulty using Zoom (eg, how to join the call or share their screen), but the bilingual research coordinator on the call was able to guide them through these issues.

Testing sessions were conducted using a semistructured interview guide (Multimedia Appendix 1) to facilitate the completion of tasks. Introductory questions were used to establish a connection with the participant before user testing of the dashboard. During the testing portion of the interview, participants were introduced to the dashboard at the time of testing so that first impressions could be observed. This also allowed for genuine reactions as they became familiar with the platform and navigated the tools. Participants were then asked to complete four major tasks, as laid out by the interview guide: (1) enter daily goal completion and review their weekly progress, (2) enter the child’s height and weight and review a growth curve, (3) contact their health care team, and (4) find resources for their family. Families were also asked about specific design layouts using preference testing (ie, participants were shown 2 design options and asked to pick the one they preferred). Throughout the testing, participants were encouraged to Think Aloud or verbalize their thoughts as they navigated independently through the tasks. The participants shared their screens with the research coordinator, which allowed the coordinator to see their navigation when using the app. Immediately following the testing portion, participants were asked closing questions to collect additional general feedback about the dashboard. Each session lasted 30 to 60 minutes.

A 3-phased approach was used to refine the dashboard and validate that iterative changes were acceptable. In phase 1, Think Aloud sessions with 4 Spanish-speaking families were conducted until no new feedback was received. The research and Nemours app team met to discuss feedback, and rapid changes were made to the user interface based on participant feedback. In phase 2, Think Aloud sessions with 3 English-speaking families were completed with the revised prototype until no substantive changes were recommended. In phase 3, a final prototype based on all of the Think Aloud feedback was shown in a video call to the larger study’s Council of Parent Partners, which was started after user testing was complete. The Council of Parent Partners included 4 parents from phase 1, one parent from phase 2, and one parent who did not participate in Think Aloud testing. This meeting was not formal user testing. Instead, it presented an opportunity to obtain final approval from parents, including many who had participated as individuals in the user testing. Meetings were facilitated by a bilingual research coordinator, with whom the parents had developed a rapport, and participants received an additional US $50 gift card for participating. Parents were advised during the group meeting that they could also provide feedback on the final prototype privately if desired.

### Data Analysis

All Think Aloud transcripts were uploaded into Dedoose [25], a qualitative data analysis software, to complete a formal qualitative analysis. A preliminary codebook of a priori codes was established based on the interview guide. The goal of the codebook was to capture the usability, understandability, and acceptability of the dashboard and its specific features during the Think Aloud sessions (Multimedia Appendix 2). Furthermore, 3 members of the research team (TLP, APR, and AO) met regularly to review the transcripts and codebook. The first transcript was coded by LP and APR together to test the codebook. All 3 members then independently coded 2 more transcripts. The team met to review discrepancies and revise the codebook as needed. The fourth transcript was used to test reliability among the coders and the established codebook. Given the strong intercoder reliability (83.5%), the codebook was finalized, and then all the transcripts were coded with the final codebook by APR and TLP. They met regularly to resolve any discrepancies. After all transcripts were coded, feedback for each of the 4 specific tasks and the overall app was pulled. The research team noted specific challenges and features highlighted by participants. This feedback was analyzed for each phase given that there were changes in-between phases. Quantitative data regarding the number of participants who reported overall acceptability of the dashboard and usability and understandability of each task were also extracted by coding specific mentions and counting the frequency of the codes.

### Ethical Considerations

Considerations to protect participants were taken into account when designing study procedures. Before recruitment, the Nemours IRB (#1812070) approved all study procedures. During recruitment, families were given the option to decline participation or opt out of further communication. Interested participants were asked to complete a consent form using an e-consent process. At a convenient time for the participant, the research coordinator trained in collecting informed consent, called the participant on the phone to review the full consent form. The consent form listed the study procedures and risks and benefits involved with the study. Participants were reminded that their participation was voluntary and that they could ask to withdraw at any time. Spanish-speaking participants were consented by a native Spanish-speaking research coordinator and with an approved Spanish-language form. Study procedures, such as having participants turn their cameras off at the time of the Think Aloud session and removing all identifying information from transcripts, were followed to protect the participants’ identities. All participants received a US $50 gift card after completing the Think Aloud session.

## Results

In total, 7 mothers (average age 39, SD 4.9 years) participated in Think Aloud sessions (4 in phase 1 and 3 in phase 2) and 6 mothers (average age 42, SD 4.9 years, 5 of whom participated in Think Aloud testing) participated in the Council of Parent Partners meeting (phase 3, Table 1). No additional participants were recruited given that user feedback did not identify any new usability problems after 2 rounds of testing. These sample sizes are in line with a typical usability study [22].

Overall, MICCO was accepted and liked by 100% (N=7) of the participants. Participants shared that the Healthy Lifestyle dashboard would be an easy-to-use tool to help them develop healthier habits as a family. As 1 participant described:

It is a tool that is within my reach. I can contact my son’s doctor to know what he can or cannot eat, and not have to make an appointment to go to the hospital. I can use the application to solve the doubts I have about some things that my son can or cannot get. For me, it would be an easy tool to handle as a mom.

While it was generally well-received, participants shared some challenges with specific tasks and highlighted features that were important to them, as described further in this study.

**Table 1:** Demographic information of caregivers of children with obesity from rural clinics in Delaware, participating in 3 iterative phases of Think Aloud Testing of a mobile dashboard to promote healthy lifestyles.

Characteristics			Phase 1 (n=4)		Phase 2 (n=3)		Phase 3^a^ (n=6)
Parent age in years, mean (SD)			38.8 (3.0)		40.3 (7.6)		42.0 (4.9)
Parent sex (female), n (%)			4 (100)		3 (100)		6 (100)
**Race, n (%)**							
	White	2 (50)		1 (33)		2 (33)	
	Black	—^b^		—		—	
	Other^c^	2 (50)		1 (33)		3 (50)	
	More than 1 race	—		1 (33)		1 (17)	
Ethnicity (Hispanic or Latino), n (%)			4 (100)		2 (67)		6 (100)
Insurance status (public)^c^, n (%)			4 (100)		2 (100)^d^		5 (83)

^a^Five participants also participated in phases 1 and 2.

^b^Not applicable.

^c^“Other” was not specified by participants.

^d^Missing n=1; public insurance refers to a program run by the government to pay for some or all health care costs.

### Phase 1

#### Overview

**Figure 2:**
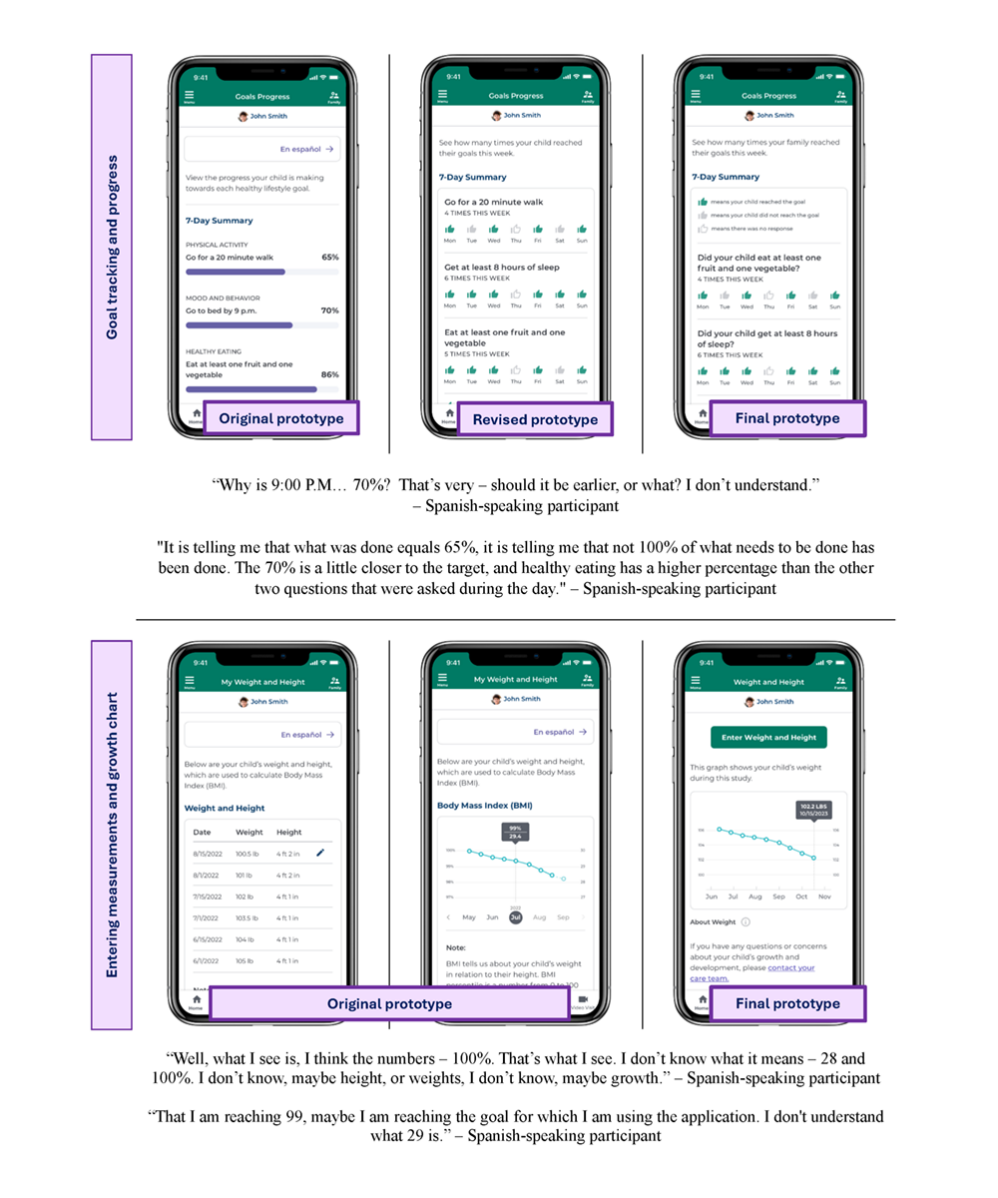
Revisions to dashboard to address numeracy challenges identified by 4 Spanish-speaking caregivers of children with obesity from rural clinics in Delaware during phase 1 of Think Aloud testing of a mobile app to promote healthy lifestyles.

**Figure 3:**
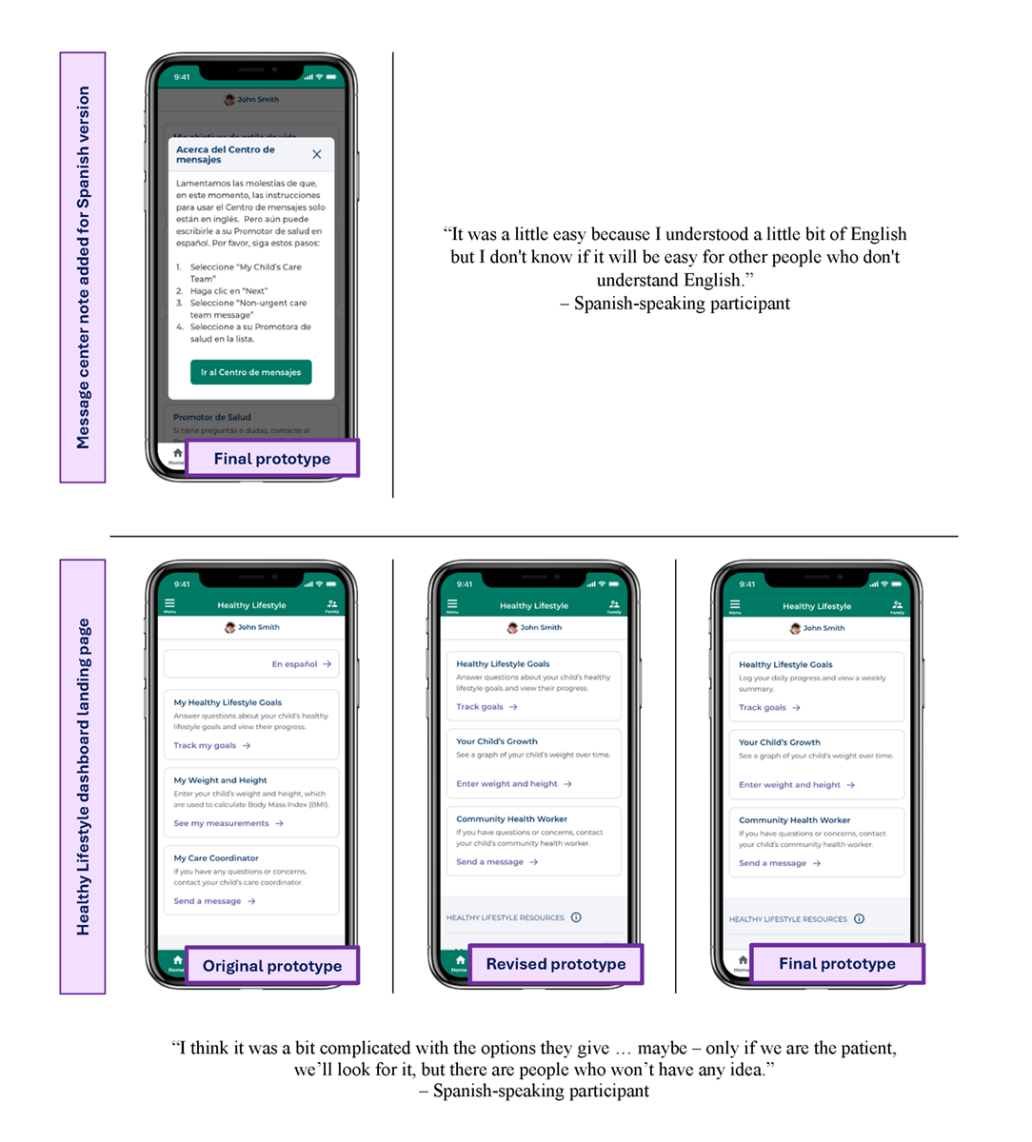
Revisions to address literacy challenges experienced during phase 1 Think Aloud testing of a mobile app to promote healthy lifestyles by Spanish-speaking caregivers of children with obesity from rural clinics in Delaware.

Spanish-speaking participants reported challenges surrounding understandability and usability of certain features of the Healthy Lifestyle dashboard. For example, 3 participants did not understand the lifestyle goal progress feature, 4 did not understand the growth curve feature, and 3 had trouble using the tool to communicate with the health care team. Themes surrounding numeracy and literacy challenges (Table 2) emerged and specific changes were made to the dashboard to ease these challenges after phase 1 (Figures 2 and 3, respectively).

**Table 2:** Challenges and solutions identified in Think Aloud testing of a mobile dashboard to promote healthy lifestyles by Spanish-speaking caregivers of children with obesity from rural clinics in Delaware.

Area and challenge		Solution
**Numeracy challenges**		
	Interpretation of weekly goal completion	Changed the percentage bar to thumbs-up icons and provided a count instead of percentage
	Interpretation of child’s growth chart	Changed growth chart to show weight change over time instead of BMI
**Literacy challenges**		
	Some components of the Nemours app (ie, the message center) were not available in Spanish	A pop-up note was added to provide the user with instructions in Spanish and a note that the following page would only be available in English
	Despite the dashboard being in Spanish, there were concerns about the reading level	Language was simplified throughout the app

#### Numeracy Challenges

All 4 participants reported numeracy issues when reviewing the lifestyle goal progress and growth curve in the dashboard. In particular, some of the participants did not understand how to interpret the percentage of goals reached over the past 7 days and some interpreted it as a “grade” or “score.” To simplify the weekly summary and ensure it would not be seen as punitive, the percentages were removed and substituted with a weekly view that used a green thumbs-up icon to indicate completion of the goal for that day, a grey thumbs-up if the goal was not completed, or a white thumbs-up if there was no data entered for that day. In the first iteration of the growth curve, participants were shown their child’s BMI over time. Despite having a note that explained how BMI was calculated and what it meant, all 4 participants struggled with the interpretation of the growth curve. Given this, the growth curve was simplified to show weight instead of BMI and to have the entry of height and weight appear on the same page as the weight curve.

#### Literacy Challenges

While all of the content in the Healthy Lifestyle dashboard was available in Spanish, there were some features of the overall Nemours app that were only available in English at the time of testing. This created some challenges. For example, participants were asked to send a message to their community health worker during Think Aloud testing. Since the Nemours app message center was only available in English, the language barrier caused some difficulty for participants. To provide families with more guidance when using the message center, a note in Spanish was added to the revised prototype to provide instructions on how to use the message center. In addition to this messaging task, 2 participants shared concerns about language barriers. As 1 participant stated:

For example, I have my mom … she definitely has trouble with technology. One, because she doesn’t really work with technology, and secondly, because there is a language barrier. Even when things are in Spanish, she still feels uncomfortable.

In recognition of these barriers, changes were made to simplify the language throughout the Healthy Lifestyle dashboard.

### Phase 2

#### Overview

**Figure 4:**
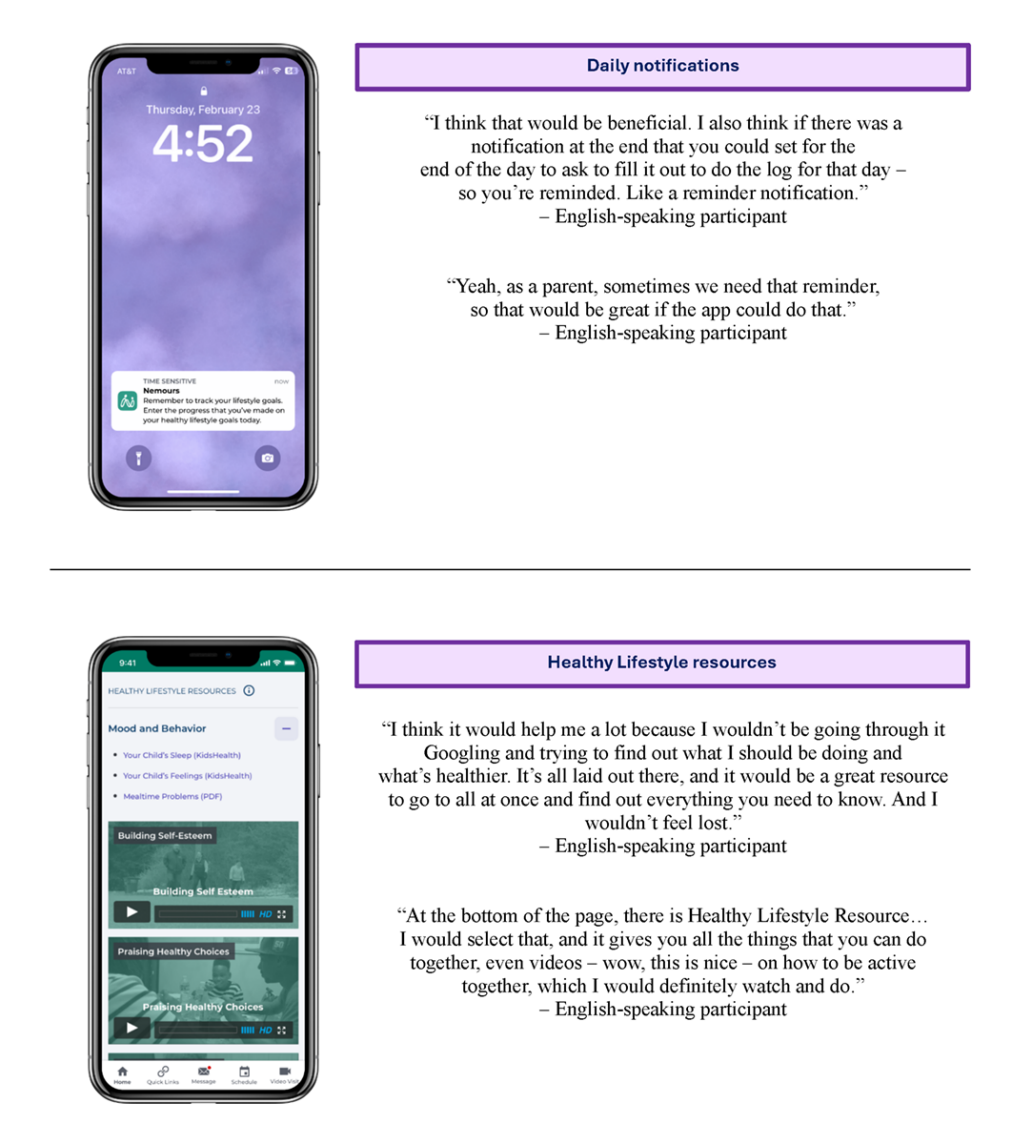
Features that caregivers of children with obesity from rural clinics in Delaware considered important to include during Think Aloud testing of a mobile app to promote healthy lifestyles.

In phase 2, participants were shown a revised version of the Healthy Lifestyle prototype based on the feedback from phase 1. New changes were well-received by parents and 3 reported that the revised prototype was usable and understandable. Feedback during this phase highlighted the importance of certain features in the dashboard (Figure 4), with only minor suggestions provided.

#### Important Features to Include

Participants liked many of the features already included in the Healthy Lifestyle dashboard and thought they would help motivate families. Participants found the multimedia resources (ie, videos, handouts, and links to external sources) embedded in the dashboard to be particularly useful. Participants especially liked the videos and thought they were a user-friendly way to view the resources. Having a set of curated resources in 1 centralized location was also an advantage because participants found it challenging to find reliable, evidence-based resources on their own. As 1 participant commented:

I would definitely promote all the things that app can help with – not just that it’s about healthy living, healthy eating, and things like that, but also that it has resources for everything, because every family’s different, and they deal with different things and different things within their children. There are great resources that you guys have and that you guys are offering.

Finally, while participants liked the lifestyle goal feature and understood the importance of tracking their lifestyle goals, many participants talked about competing priorities that may get in the way of inputting their data. As a solution, they discussed how an evening reminder to log their goals would help keep them on track.

#### Communication and Integration With Health Care Team

All participants discussed the importance of being able to communicate with their health care team through the dashboard, whether by sending messages or by having lifestyle goals and weight data available to their health care team. One participant highlighted how the transfer of the dashboard data to the child’s care team could help keep families accountable for their goals:

I also think maybe to some parents, it would also be motivating to know – like if this is going to the provider, you want to show that as a parent, you’re being proactive, and you’re doing what you need to do for your child.

Despite the appeal of communicating with their health care team through the app, participants expressed a need for more clarity about who would view their data and how and when the health care team would be communicating back to them, which was incorporated into the final prototype.

#### Preference Testing

**Figure 5:**
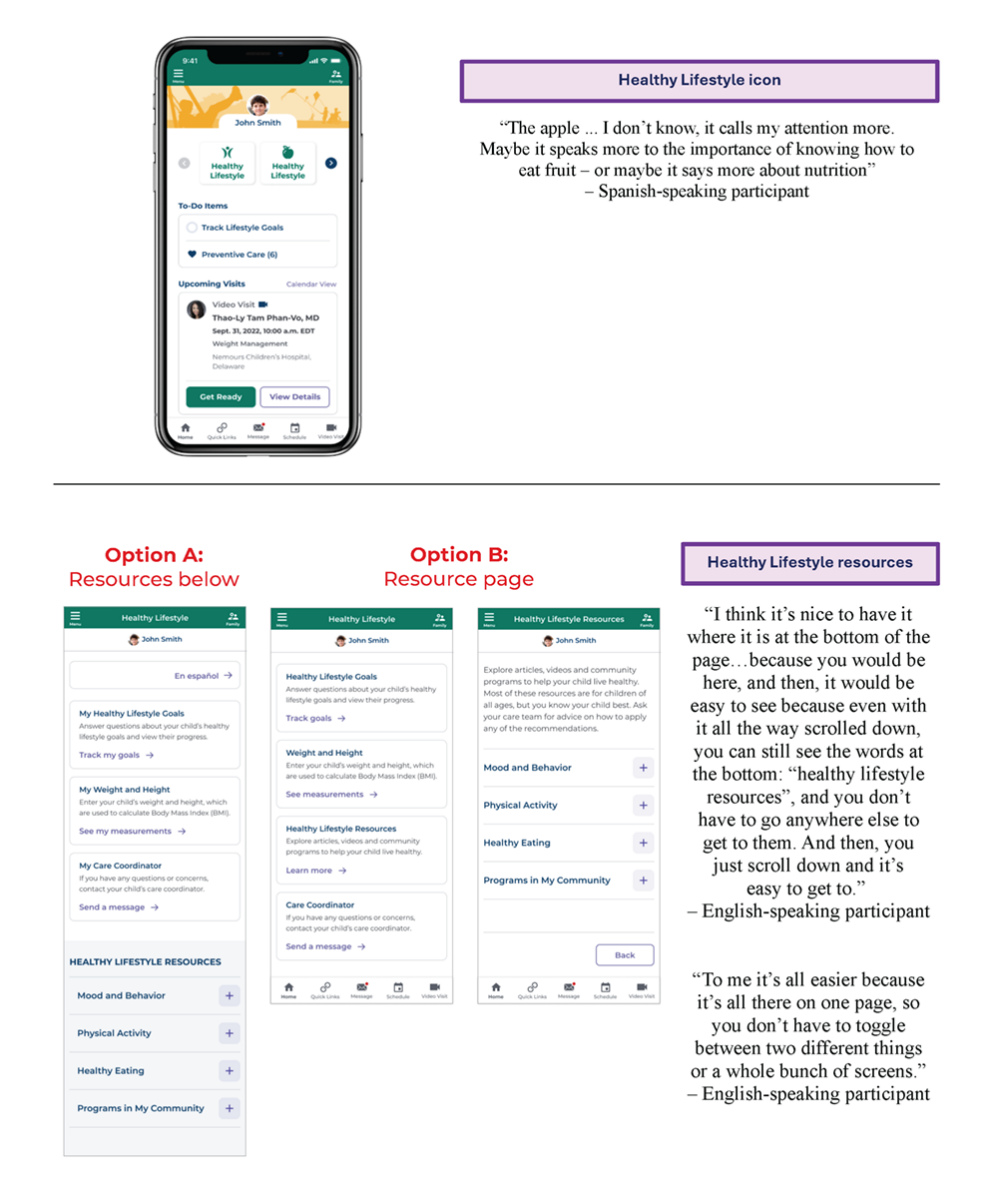
Results of preference testing done during Think Aloud testing of caregivers of children with obesity from rural clinics in Delaware to determine a dashboard icon and resource page layout for a mobile app to promote healthy lifestyles.

Preference testing is a research method during which participants compare 2 variations of a design, choose 1, and explain their preference [21], and it was used in both phases 1 and 2. This method was used for selecting an icon to represent the Healthy Lifestyle dashboard and determining the preferred way to incorporate resources (Figure 5).

#### Healthy Lifestyle Dashboard Icon

Since the Healthy Lifestyle dashboard is incorporated into the Nemours app, which is a much broader app designed for a range of health care needs, an icon was needed to launch the dashboard. Designers on the Nemours app team explored a range of concepts for the icon. Symbols that represent different aspects of a healthy lifestyle, like physical activity and nutrition, were considered. The top 2 designs—a joyous person (left) and an apple (right)—were shared during phase 1 testing. Based on feedback from all the participants, the apple icon was chosen to be the branding element for the Healthy Lifestyle dashboard. Participants cited liking the apple more because they associated it with nutrition, which was the primary domain that participants associated with healthy lifestyles.

#### Resource Page Layout

The list of Healthy Lifestyle resources included 19 items across 4 domains (healthy eating, being active, mood and behavior, and community programs). Preference testing was used to understand how participants preferred to access the resources. In total, 2 variations of the Healthy Lifestyle resources were designed. Option A made the resources available at the bottom of each page (like a footer) with expandable sections. Whereas option B involved adding them to the dashboard as a fourth link, which would open a new page for this section. Participants compared the 2 options and then indicated their preference. Though 2 of the 7 participants thought option B looked better, the majority picked option A. They liked having all the resources easily accessible on every page instead of having to navigate back and forth through the pages.

### Phase 3

All 6 parents (refer to Table 1 for demographic information) who participated in phase 3 were in agreement that the revisions to the dashboard addressed their concerns from phase 1 or 2. They reported that the final prototype was useful and easy to understand. Given no further feedback about the dashboard, it was finalized for use in future research studies.

## Discussion

### Principal Findings

MICCO is a comprehensive, multicomponent intervention that was developed in collaboration with community, parent, and health care partners to improve the relevance and accessibility of pediatric obesity interventions for families from underserved and rural communities. Digital self-management tools are a key component of the MICCO intervention and are made available to families through a mobile app dashboard integrated with the EHR. This dashboard component of MICCO addresses notable limitations in the mHealth for childhood obesity literature by including community resources, being available in Spanish, incorporating culturally relevant resources, and being integrated into health care delivery. Human-centered design methods were applied to ensure that the needs and preferences of families of diverse backgrounds were incorporated. The resulting dashboard is user-friendly, literacy-sensitive, and culturally responsive and incorporates features that will facilitate its usability in future research and clinical practice.

Through a human-centered approach to design, the research team was able to better understand the challenges that families, especially those with limited English proficiency and from rural neighborhoods, may experience in using an app to facilitate healthy lifestyle behaviors. Challenges navigating health information are not unique to this study and have been described among larger cohorts of Spanish-speaking individuals of Hispanic or Latino ethnicity [26,27]. Even when health information is available in Spanish, studies have found that reading levels are typically above a sixth-grade level which is higher than the recommended reading level for health information in any language [28,29]. Difficulty understanding health information can contribute to low rates of engagement with programs and services among Spanish-speaking populations [27,30,31]. Therefore, testing mHealth interventions with Spanish-speaking users is critical to ensuring that the intervention is understood and well-received by these populations.

In this study, numeracy and literacy barriers were identified during Think Aloud testing with Spanish-speaking participants. Therefore, pages depicting weight and goal progress were simplified to convey relevant health information more clearly. In addition, information was added to help Spanish-speaking families navigate the 1 component (messaging function) of the app that was not fully available in Spanish at the time of testing. Not only were these adaptations made as part of this research study, but findings from this line of research in conjunction with other Diversity, Equity, and Inclusion initiatives at Nemours Children’s Health, prompted an overhaul of the entire Nemours App to be fully available in Spanish after testing.

Feedback received during usability testing also helped the team develop a deeper understanding of families’ busy lifestyles. In particular, the majority of participants expressed challenges balancing competing priorities when taking care of young children. Not surprisingly, time constraints are a commonly cited barrier to adherence in childhood obesity treatment [32,33]. Because of this, many participants reported that an evening reminder about tracking lifestyle goals would be helpful, so a push notification was implemented to remind families to track their lifestyle goals in the app. Participants also liked the curated resources available through the dashboard, especially the brief videos. Having resources that were easily accessible added to the convenience of the app for families; in addition to automated reminders, this may be a way to keep families engaged in treatment.

Finally, participants endorsed an appreciation for the integration of the dashboard with their overall health care. For example, they reported a desire for information tracked through the dashboard to be shared with their health care team and wanted to know more about how to communicate with their health care team. The Healthy Lifestyle dashboard was designed to facilitate this bidirectional communication. This approach is different than most apps designed for childhood obesity, which are stand-alone. While there are advantages to this, findings from this study suggest that mHealth interventions may be more effective if integrated with health care delivery, especially for rural communities that may experience lower access to health care facilities. Not only can this enhance satisfaction and motivation on the part of families, but it can also inform clinical decision-making on the part of the health care team [34,35].

### Limitations

There were some limitations to this study. While the recruitment strategy was broad and a diverse sample was recruited, it is possible that our sample was biased toward those with more digital literacy and broadband access. In the future, it will be important to evaluate the app across families from varying socioeconomic backgrounds and with those who face greater barriers to care. Using in-person testing may also allow for testing of patients with limited digital access and provide a better understanding of nonverbal cues that may not have been captured in the video recording. While our sample size was small, it is consistent with best practices for usability testing but there may be limitations in our methods of obtaining final input through an Advisory Council (vs repeated formal usability testing). Also, while participants endorsed the acceptability of the dashboard, the study’s focus on usability does not allow for an understanding of whether participants would engage with it on a routine basis. A future study might conduct field testing of the MICCO app, wherein participants can use the app continuously for a shortened duration (eg, a month) and provide acceptability and usability feedback before it is deployed in a longer trial. In addition, previous studies have identified that digital interventions are subject to low uptake and engagement levels; therefore, further research using implementation frameworks will be needed to better understand factors that will facilitate routine use of the dashboard as part of the larger intervention. Finally, while the dashboard (and intervention more broadly) was designed for caregivers of young children, future studies should consider refining and testing the dashboard for youth with obesity who may find a digital intervention even more acceptable.

### Conclusion

Collaborating with community and patient partners and conducting usability testing with key demographic populations, especially Spanish-speaking populations, was important to developing an mHealth intervention that is user-friendly, culturally relevant, and literacy-sensitive to promote pediatric healthy lifestyles among diverse families. Designing an intervention that is usable for intended users and in intended settings is a critical first step to trialing and implementing a successful intervention.

## Appendix

Multimedia Appendix 1 Mobile Integrated Care for Childhood Obesity (MICCO) interview guide used to guide Think Aloud sessions with caregivers of young children with obesity.

Multimedia Appendix 2 Mobile Integrated Care for Childhood Obesity (MICCO) – Think Aloud code book.

## Abbreviations

EHR: electronic health record
mHealth: mobile health
MICCO: Mobile Integrated Care for Childhood Obesity

## Data Availability

Deidentified data are available upon reasonable request. All requests should be directed to the corresponding author.
