# Supplementary material for: mHealth App to Promote Healthy Lifestyles for Diverse Families Living in Rural Areas: Usability Study
Source: JMIR Form Res. 2025 Feb 11;9:e60495. doi: 10.2196/60495 (PMC11862780; doi:10.2196/60495)
Supplement: Multimedia Appendix 1 [file formative_v9i1e60495_app1.docx]

*Introduction*

Hi, thank you for taking the time to meet with me to test our new Healthy Lifestyles App. As a reminder, you will be asked you to use different parts of the App and describe everything you are thinking and doing during this session. At the end of the Think Aloud Testing, we will ask you a few questions about whether you liked the App and found it helpful. You will receive a $50 dollar gift card after completing the session.

This session should be 30-60 minutes long and will be recorded. We will delete any personal information from our transcript of the recording and will delete the recording after the study is complete. As a reminder, this is completely voluntarily so we can stop at any point, and you don’t have to answer any question that you are uncomfortable with.

- Do you still agree to be in this study?
- Do you have any questions for me before we start?

Alright, so with your permission, I will go ahead and start recording. Please turn your camera off (if on). And I am just going to say something into the recording for my records before we start with the questions. ***Hit Record***

**Interview:**

The recording has started. Today’s date is [Date] and I am here with participant # X.

**Section A: Tell me a little bit about your family**

1. **How has your experience been helping your child with healthy lifestyle behaviors?**
   1. What resources and strategies have you found to be most helpful?
   2. What has been the most challenging thing?

Thank you for sharing that with me! Now we are going to move onto reviewing the App. I have sent you a link to the App. Please click on the link and open it up in your internet browser on your device.

**Section B: Think Aloud Test**

***Introduction***

We are now going to complete the “Think Aloud” portion of this session. During this portion of the session, I am going to let you explore the App and complete a series of tasks on your own. We are not testing you or your knowledge about healthy lifestyles. We’re testing to how easy or hard it is to use the App. Keep in mind, some parts of the App will not be work because this is not the final version and we are interested in getting feedback about certain parts.

As you are going through the App, I am going to ask that you share your thoughts out loud.

For example, if you are trying to figure out how to go back to the home page, you may say something like, “Right now, I want to go to the home page because I have finished answering questions about my child’s lifestyle goals. I am looking for the button that will take me back home, but I am not sure where to find it. Oh, I see it now. It was kind of hard to find because the button is small. Okay. I am back home”

Hearing these thoughts will allow us to see how easy or hard it is to use the App. You may also share thoughts about what you think about the different contents of the App you are seeing.

I may give you some reminders as you are going through the App to say your thoughts out loud.

Before we get started, do you have any questions?

***Scenario***

Like I mentioned at the beginning, I am going to ask you to complete some tasks in the App. We are going to walk through the following scenario:

You and your child had a visit with a Nemours Healthy Weight and Wellness provider. During the visit, you discussed 3 lifestyle goals for your child.

1. Go for a walk for 20 minutes
2. Go to bed by 9pm
3. Eat a least one fruit and one vegetable

At the end of the visit, they also mentioned to you that there is a new Healthy Lifestyle program that can be found on the Nemours App where you can keep track of your child’s goals and weight. You can also find resources to help your family implement healthy lifestyles.

**Task 1: Opening the App**

You get home, open your Nemours App.

You want to explore the Healthy Lifestyle program. Can you show me how you would do this? Remember to describe what you’re doing.

- *Where did your eyes go first?*
- *What are you looking for now?*
- *Where are you going to click next?*
- *Did you notice there are two icons? Why did you select the one you did?(****Phase 1****)*

**Task 2: Tracking Goals**

Great! You are now inside the program.

- *Where did your eyes go first?*

As I mentioned in the beginning, during your visit, you discussed 3 goals for your child at your visit. You want to mark that your child completed these goals on September 1, 2022. Can you show me how you would do this? Remember to describe what you’re doing.

*Associated tasks:*

- *Clicking on the button “Healthy Lifestyle Goals”*
- *Entering date*
- *Checking yes to each question*
- *Saving entry*
- *Viewing 7-day goal summary -What are you seeing on this page? How would you interpret this visual?*

Follow-up Question:

- How helpful do you think it would be to include daily app notifications to complete the daily log with your progress for each goal?
  - If helpful, is there a specific time you would like to receive the reminder?

**Task 3: Measurements**

Great! Now, you want to enter your child’s height and weight. Can you show me how you would do this? Remember to describe what you’re doing.

***Phase 1***

*Associated tasks:*

- *Clicking on the button “Height and Weight”*
- *Clicking on the button to have the scale measure weight*
- *Manually entering height*
- *Clicking on information icon as needed to learn how to use the scale and ruler*
- *Viewing the BMI graph.*

Follow-up Question:

- What do the numbers on the weight tracking graph mean to you? How helpful is it to display BMI on this graph?

***Phase 2 (Questions were revised to reflect the new design based on changes made after Phase 1)***

*Associated tasks:*

- *Clicking on the button “Your Child’s Growth”*
- *Viewing the weight graph.* How would you interpret this visual?
- *Clicking on the button “Enter Weight and Height”*
- *Clicking on the button to Use scale*
- *Manually entering height*
- *Clicking on information icon as needed to learn how to use the scale and ruler*
- *Saving entry*

**Task 4: Community Health Worker/Care Coordinator**

Great! You are trying to plan your meals with your child and realize you want to know if a tomato is considered a fruit or a vegetable. Your community health worker/care coordinator said you could contact them to ask them any questions. Can you show me how you would do this? Remember to describe what you’re doing.

*Associated tasks:*

- *Clicking on the button “Community Health Worker”/ “Care Coordinator”*
- *Selecting the community health worker to message*
- *Adding a subject and text*
- *Clicking send*

**Task 5: Accessing Resources**

Great! Finally, you want to learn more about how to help your child to be more active at home. Can you show me how you would do this? Remember to describe what you’re doing.

*Associated tasks:*

- *Clicking to expand the resource section*
- *Finding section on physical activity*
- *Clicking on links and videos (does not necessarily have to view links or videos)*

Follow-up Question:

- What do you think about having the resources on a separate page versus having them on the bottom of the activity page?

Great! Those are all the tasks I had for you. Are there any other features of the App you would like to explore before we move onto the last section of the interview?

**Section C: Debrief and overall thoughts about the App**

Great! Thank you for going through the App. Before we finish today, I just want to ask you a few extra questions about the App.

1. **What is your overall impression of the app?**
   1. What did you like best/least about the App?
   2. What would make you more/less likely to use the App?
   3. What barriers would you have or think other people might have to using the App (e.g. no internet access, no time, no interest)?
2. **How useful did you find the App?**
   1. How much do you think the resources and tools in the App would help your child and your family?
   2. How would you see yourself using the App outside of this study? How often would you use it?

Thank you for sharing that with me!

**Section D: Closing**

1. **What else would be helpful for our team to know before we share this app with other parents and caregivers?**

Thanks! That’s all I have for today. I will turn off the recording now.

I am just going to confirm your mailing address, so I know where to send the gift card. As a reminder, at Nemours, we use a gift card company called Greenphire. We will share your name, date of birth, and mailing address with them to load your gift card with money.
