# Supplementary material for: mHealth App to Promote Healthy Lifestyles for Diverse Families Living in Rural Areas: Usability Study
Source: JMIR Form Res. 2025 Feb 11;9:e60495. doi: 10.2196/60495 (PMC11862780; doi:10.2196/60495)
Supplement: Multimedia Appendix 2 [file formative_v9i1e60495_app2.docx]

| **Code #** | **Code Name** | **Definition** | **Subcodes^a^** |
| --- | --- | --- | --- |
| **1.0** | Experiences with Healthy Lifestyle | Use when participant describes how their family lives/tries to live a healthy lifestyle  Use subcode (**1.1**) when participant shares specific activities/habits they do to live a healthy lifestyle  Use subcode (**1.2**) when a participant shares challenges with living a lifestyle  Double code (**1.3-1.5**) when a participant discusses a particular aspect of healthy lifestyles  Use subcode (**1.6**) when a participant discusses why it is important to be healthy. | - - 1.1 Current Habits   - 1.2 Challenges   - 1.3 Nutrition   - 1.4 Physical activity   - 1.5. Psychosocial (sleep, behaviors, etc.)   - 1.6 Importance of health |
| **2.0** | App Usability | Use to describe participant’s feedback about navigating the App (or an App feature)  May be double coded with specific App function code (**6-9**) if feedback is specific to a certain function/section within the App | - 2.1 Easy - 2.2 Challenging - 2.3 Neither easy nor challenging |
| **3.0** | App Acceptability | Use when participant discusses whether they like the App (or an App feature)  May be double coded with specific App function code (**6-9**) if feedback is specific to a certain function/section within the App  Use subcode (**3.3**) when participant shares thoughts about barriers one may face to using the App | - 3.1 Like - 3.2 Dislike - 3.3 Barriers - 3.3.1 Technology - 3.3.2 Competing priorities/busy - 3.3.3 Literacy/language barriers - 3.3.4 Motivation - 3.4 Neither like nor dislike - 3.5 Important features to include |
| **4.0** | App Understandability | Use when participant provides feedback about whether App (or an App feature) content is understandable  May be double coded with specific App function code (**6-9**) if feedback is specific to a certain function/section within the App. | - 4.1 Understandable - 4.2. Not understandable - 4.3. Neither understandable nor not understandable - 4.4 Numeracy challenges |
| **5.0** | Wording | Use when participant shares feedback about specific wording/phrasing being used in the app  May be double coded with specific App function code (**6-9**) if feedback is specific to a certain function/section within the App.  Use subcodes (**5.1; 5.2**) if wording suggestion is for English or Spanish version of the App, respectively | - 5.1 English - 5.2 Spanish |
| **6.0** | Tracking Lifestyle Goals function | Use when participant shares feedback about the Tracking Lifestyle Goals function  Use subcodes (**6.1; 6.2; 6.3**) when talking about specific features in the Tracking Lifestyle Goals function | - 6.1 Entering lifestyle goals - 6.2 Reviewing 7-Day Summary - 6.3 Receiving notifications |
| **7.0** | Entering Measurements function | Use when participant shares feedback about Entering Measurements function  Use subcodes (**7.1; 7.2; 7.3**) when talking about specific features in the Entering Measurements function | - 7.1 Entering new measurement - 7.2. Using scale - 7.3 Reviewing growth chart |
| **8.0** | Contacting Community Health Worker function | Use when participant shares feedback about contacting the care coordinator  Use subcodes (**8.1; 8.2**) when talking about specific features in the Contacting Community Health Worker function | - 8.1 Selecting community health worker - 8.2. Entering and sending message |
| **9.0** | Accessing Resources function | Use when participant shares feedback about Resources function  Use subcodes (**9.1; 9.2**) when talking about specific features in the Resources function | - 9.1 Finding resource section - 9.2 Finding specific resource |
| **10.0** | Formatting of Resources (A/B Testing) | Use subcodes (**10.1; 10.2**) depending on option chosen during A/B Testing | - 10.1 A-Option - 10.2 B-Option |
| **11.0** | App Icons (A/B Testing) | Use subcodes (**11.1; 11.2**) depending on option chosen during A/B Testing | - 11.1 Person - 11.2 Apple |

1. Subcodes are independent of one another and can be double-coded with other subcodes and codes if applicable
